# Development of a Clinical Forecasting Model to Predict Comorbid Depression Among Diabetes Patients and an Application in Depression Screening Policy Making

**DOI:** 10.5888/pcd12.150047

**Published:** 2015-09-03

**Authors:** Haomiao Jin, Shinyi Wu, Paul Di Capua

**Affiliations:** Author Affiliations: Shinyi Wu, PhD, University of Southern California and RAND Corporation, Los Angeles, California; Paul Di Capua, MD, MBA, University of California, Los Angeles, Los Angeles, California.

## Abstract

**Introduction:**

Depression is a common but often undiagnosed comorbid condition of people with diabetes. Mass screening can detect undiagnosed depression but may require significant resources and time. The objectives of this study were 1) to develop a clinical forecasting model that predicts comorbid depression among patients with diabetes and 2) to evaluate a model-based screening policy that saves resources and time by screening only patients considered as depressed by the clinical forecasting model.

**Methods:**

We trained and validated 4 machine learning models by using data from 2 safety-net clinical trials; we chose the one with the best overall predictive ability as the ultimate model. We compared model-based policy with alternative policies, including mass screening and partial screening, on the basis of depression history or diabetes severity.

**Results:**

Logistic regression had the best overall predictive ability of the 4 models evaluated and was chosen as the ultimate forecasting model. Compared with mass screening, the model-based policy can save approximately 50% to 60% of provider resources and time but will miss identifying about 30% of patients with depression. Partial-screening policy based on depression history alone found only a low rate of depression. Two other heuristic-based partial screening policies identified depression at rates similar to those of the model-based policy but cost more in resources and time.

**Conclusion:**

The depression prediction model developed in this study has compelling predictive ability. By adopting the model-based depression screening policy, health care providers can use their resources and time better and increase their efficiency in managing their patients with depression.

## Introduction

Clinical forecasting analyzes current and historical facts to predict clinical outcomes. Such forecasting has important applications for underdiagnosed conditions such as comorbid depression among patients with diabetes (1,2), who are twice as likely to suffer depression as the general population (prevalence, 10%–15%) (3,4). For approximately 45% of patients with diabetes, depression goes undiagnosed (3). Mass depression screening improves diagnosis rates (5) but requires significant resources, which prevents providers (6), especially providers in resource-constrained safety-net clinics (7), from adopting this screening method. Providers could screen only diabetes patients at high risk of depression, but the complex relationships between depression and its risk factors make it difficult to identify only patients at high risk (8).

Machine learning methods can automatically detect patterns in data and use the patterns to predict future data (9). Machine learning is related to statistics but emphasizes individual-level prediction rather than population-level inference (10). Machine learning was used to develop prediction models for outcomes such as mortality (11,12) and depression (13–15). The objectives of our study, Predicting Diabetes Patients with Comorbid Depression (PreDICD), were 1) to apply machine learning methods to developing an individual-level clinical forecasting model by using diabetes care-related predictors that are easy to acquire or are recommended in clinical practice and 2) to evaluate a model-based screening policy that assigns depression screening only to patients predicted as being depressed by the model. Such a model could save time and resources by not screening patients predicted as nondepressed unless warranted by further model forecasting or clinical observation.

## Method

We developed the PreDICD model by using machine learning methods. Then, we compared the model-based screening policy with mass screening to evaluate the policy’s influence on provider resources and time and on the rate of depression identification. We also compared the model-based policy with 3 heuristic-based partial screening policies that assign depression screening to patients with certain risk factors (including depression history or severe diabetes or both) and assessed the implications for provider’s choice of depression screening policy.

### Depression measure

The study measured depression by using Patient Health Questionnaires PHQ-9 and PHQ-2, well-validated tools for depression screening (16,17). PHQ-9 consists of 9 questions that are the same 9 criteria used for the diagnosis of depressive disorders as defined by the *Diagnostic and Statistical Manual of Mental Disorders*, *Fourth Edition* (*DSM-4*). Each question has 4 ordinal responses with assigned scores from 0 to 3; therefore, the overall scale has a possible score from 0 to 27, where the higher scores indicate more severe depression. PHQ-2 consists of the first 2 PHQ-9 questions. PHQ-2 often serves as a fast screening tool (16, 17); a score of 3 or higher on PHQ-2 warrants a PHQ-9 evaluation to formally diagnose a depressive disorder or assess severity of depression (17). Major depression (the predicted outcome in this study) is indicated by a PHQ-9 score of 10 or higher. Validity of this cutoff point has been established by Kroenke, Spitzer (16).

### Data set

Data used to develop the PreDICD model were obtained from 2 clinical trials with underserved, predominantly Hispanic, patients with diabetes: the Diabetes-Depression Care-Management Adoption Trial (DCAT) and the Multifaceted Diabetes and Depression Program (MDDP). DCAT is a comparative effectiveness study conducted from 2010 through 2013 in safety-net clinics in the Los Angeles County Department of Health Services (LACDHS), the second largest safety-net healthcare system in the United States. DCAT tested an automated telephone depression screening and monitoring system integrated with a collaborative care management program to facilitate adoption of a collaborative depression care model (18,19). MDDP is a randomized trial conducted from 2005 through 2009 testing the collaborative depression care model for underserved LACDHS patients with comorbid depression and diabetes. The 2 trials are described elsewhere (5,19).

The combined data sets provided the important benefit of balancing the proportions of depressed (PHQ-9 score ≥10, 43.8%) and nondepressed patients (PHQ-9 score <10, 56.2%). In a prior analysis (20), we investigated the use of DCAT data alone to predict depression. Because the nondepressed instances dominated over depressed instances in the DCAT data, the derived model was easily overfitting to the nondepressed instances. The balanced proportions of depressed and nondepressed patients can help the PreDICD model avoid overfitting to either nondepressed or depressed instances and thus improve the predictive ability of the model.

### Candidate predictors and predictor selection

We identified 20 candidate predictors from the combined DCAT–MDDP data in accordance with 2 criteria: 1) the candidate predictors were relevant to diabetes care and measure aspects that were supported by 2 prior systematic reviews (4,8) as being correlated with depression, and 2) the candidate predictors were typically obtainable from electronic medical records (EMR) or were recommended for providers to routinely collect during diabetes clinic visits. The 20 candidate predictors are summarized in Table 1. They included common demographics, diabetes characteristics, depression history, other health conditions, and level of health care use.

**Table 1:** Data on Patients (N = 1,793) Served by Los Angeles County Safety-Net Clinics, DCAT (2010–2013) and MDDP (2005–2009), Used to Train and Validate the PreDICD Prediction Model

Parameter	Patients from DCAT		Patients from MDDP		*P* c	Patients From the Combined Data Set	
	N^a^	Statistics^b^	N^a^	Statistics^b^		N^a^	Statistics^b^
**Depression symptoms**							
PHQ-9 (possible score: 0–27; higher = more severe depression)	1,406	6.67(6.00)	387	14.72(2.95)	<.001	1,793	8.41(6.41)
PHQ-910	1,406	399 (28.38%)	387	387 (100%)	<.001	1,793	786 (43.84%)
**Demographics**							
Age, y	1,406	53.27 (9.24)	387	53.97 (8.74)	.17	1,793	53.42 (9.13)
Hispanic/Latino	1,403	1,254 (89.38%)	387	372 (96.12%)	<.001	1,790	1626 (90.84%)
BMI	1,385	32.73 (7.28)	383	32.90 (7.55)	.69	1,768	32.77 (7.34)
Female	1,406	892 (63.44%)	387	318 (82.17%)	<.001	1,793	1210 (67.48%)
**Diabetes characteristics**							
Years with diabetes	1,379	10.27 (7.64)	385	10.32 (8.60)	.92	1,764	10.28 (7.86)
Hemoglobin A1c (%)	1,344	9.24 (2.12)	374	9.03 (2.19)	.10	1,71,8	9.19 (2.14)
Hemoglobin A1c tested	1,406	1,344 (95.59%)	387	374 (96.64%)	.36	1,793	1718 (95.82%)
Toobert diabetes self-care (range 0–7, higher=better diabetes self-care)	1,406	4.33 (1.31)	387	3.38 (1.45)	<.001	1,793	4.12 (1.40)
Total number of diabetes complications	1,406	1.27 (1.15)	387	1.45 (1.04)	.004	1,793	1.31 (1.13)
On insulin	1,406	742 (52.77%)	387	107 (27.65%)	<.001	1,793	849 (47.35%)
On diabetes oral medication	1,406	1,227 (87.27%)	387	321 (82.95%)	.03	1,793	1548 (86.34%)
**Depression history**							
Previous diagnosis of major depressive disorder	1,406	120 (8.53%)	387	74 (19.12%)	<.001	1,793	194 (10.82%)
**Other health conditions**							
Previous diagnosis of panic	1,406	7 (0.50%)	387	5 (1.29%)	.09	1,793	12 (0.67%)
Previous diagnosis of anxiety	1,406	14 (1.00%)	387	11 (2.84%)	.006	1,793	25 (1.39%)
Number of ICD-9 diagnoses in past 6 months	1,389	7.03 (4.45)	387	7.93 (3.56)	<.001	1,776	7.23 (4.29)
Chronic pain	1,406	354 (25.18%)	387	126 (32.56%)	.004	1,793	480 (26.77%)
**Self-rated health status**							
1 (Poor)	1,406	223 (15.86%)	387	144 (37.21%)	<.001	1,793	367 (20.47%)
2 (Fair)		633 (45.02%)		206 (53.23%)			839 (46.79%)
3 (Good)		468 (33.29%)		27 (6.98%)			495 (27.61%)
4 (Very good)		69 (4.91%)		7 (1.81%)			76 (4.24%)
5 (Excellent)		13 (0.92%)		3 (0.78%)			16 (0.89%)
**Health care use**							
Hospitalization in past 6 months	1,406	218 (15.50%)	387	62 (16.02%)	.80	1,793	280 (15.62%)
Admitted to Emergency Department in past 6 months	1,404	385 (27.42%)	387	63 (16.28%)	<.001	1,791,	448 (25.01%)
Number of outpatient clinic visits in past 6 months	1,406	2.81 (3.56)	387	2.96 (2.81)	.38	1,793	2.84 (3.41)

Abbreviations: BMI, body mass index; DCAT, Diabetes–Depression Care-Management Adoption Trial; ICD-9, *International Classification of Diseases, 9th Revision*; MDDP, Multifaceted Diabetes and Depression Program; PHQ-9, Patient Health Questionnaire, 9-items; PreDICD, Predicting Diabetes Patients with Comorbid Depression.

a Number of respondents

b Values are numbers (column percentages) for categorical variables and mean (standard deviation) for continuous variables

c *P* values were calculated by using χ^2 ^test for categorical variables and *t* test for continuous variables.

From the candidate predictors we selected predictors for developing the PreDICD model. Available selection methods were variable ranking, subset evaluation, and the wrapper method (21). For this study, we adopted a correlation-based subset evaluation method developed by MA Hall (unpublished doctoral dissertation, *Correlation-Based Feature Selection for Machine Learning*. Hamilton (Waikato Region, New Zealand): The University of Waikato; 1999) that searches predictors by greedy hill-climbing algorithm and targets to select a subset of predictors that are highly correlated with the outcome measure while having low intercorrelation. This predictor selection procedure was carried out by machine learning software, Weka, version 3.6.11 (Slashdot Media).

### Model development and validation

To derive the appropriate model, we trained and cross-validated (10-fold) 2 linear machine learning models, logistic regression (with Ridge parameter to improve predictive ability [22]) and multilayer perceptron; and 2 nonlinear models, support vector machine (SVM) and random forest. Model selection was based on the 4 models’ predictive ability. The primary criterion was the area under the receiver operating characteristic curve (AUROC), where a larger AUROC indicates better overall predictive ability. We also evaluated the percentages of correctly classified instances, sensitivity, and specificity. We used the model with the best overall predictive ability, measured by AUROC, as the ultimate PreDICD model. Model validation was also carried out by Weka, version 3.6.11, and the ultimate PreDICD model was fitted by R, version 3.1.1 (https://cran.r-project.org/bin/windows/base/old/3.1.1/), by using the whole data set.

### Evaluating and comparing the model-based depression screening policy

The PreDICD model can support a model-based screening policy that assigns depression screening only to patients predicted by the model to be depressed. We compared the model-based policy with mass depression screening to evaluate the influence of model-based policy on provider resources and time and on the rate of depression identification. In addition, we compared the model-based policy to 3 heuristic-based partial screening policies used by providers to save resources and time. The first heuristic, which requires depression screening for patients with a previous diagnosis of major depressive disorder, is based on the fact that depression is a highly recurrent disease (23). The second heuristic, which requires depression screening for patients with severe diabetes (hemoglobin A1c9.0%), is based on the evidence that diabetes and depression are often comorbid conditions (3,4). The third heuristic combines the other 2, requiring patients with either a previous diagnosis of major depressive disorder or severe diabetes to be screened for depression.

We evaluated the model-based policy and compared it with mass screening and 3 heuristic-based policies under the clinical context that PHQ is used for depression screening. We assumed the scenario in which patients meeting screening policy inclusion criteria were evaluated using the 2-step PHQ screening suggested by Kroenke et al (17): PHQ-2 is first assigned, and then patients with a PHQ-2 score of 3 or higher are further evaluated by PHQ-9. We compared the rate of depression identification and 3 measures relevant to provider resources and time: proportion of patients receiving PHQ-2 screening, proportion of patients receiving PHQ-9 screening, and the number of questions asked per patient. We further evaluated and compared policies in another scenario in which the 2-step PHQ screening is bypassed in favor of the complete PHQ-9 screening for all patients meeting screening policy inclusion criteria. We compared the same measures as the first scenario.

To evaluate the model-based policy, we trained the PreDICD model on the combined DCAT–MDDP data; however, we cross-validated (10-fold) only the DCAT data. That is, we randomly divided the samples from DCAT into 10 roughly equal parts. In each single round of validation, samples from 9 of the 10 parts of DCAT data plus samples from MDDP were used to train the prediction model; we then validated the trained model on samples from the remaining data. Mass screening and the 3 heuristic-based policies were also evaluated only on the DCAT data. Because the DCAT data included data on both depressed and nondepressed patients, they represented the LACDHS safety-net population better than the MDDP data. All comparisons were 2-sided and carried out by statistical software R, version 3.1.1.

## Results

### The PreDICD model

We identified 1,793 patients from the combined DCAT and MDDP data. The MDDP trial enrolled only depressed patients with diabetes (PHQ-9 ≥10 ), and the DCAT trial enrolled both depressed (PHQ-9 score ≥10, 28.4%) and nondepressed (PHQ-9 score <10, 71.6%) patients with diabetes (Table 1). The combined sample was predominantly Hispanic with balanced proportions of depressed and nondepressed patients (PHQ-9 score ≥10, 43.8%; PHQ-9 score <10, 56.2%).

We used a correlation-based subset evaluation predictor selection method for the PreDICD model to select 7 predictors that are highly correlated with major depression and have low intercorrelation: 1) female, 2) Toobert diabetes self-care, 3) total number of diabetes complications, 4) previous diagnosis of major depressive disorder, 5) number of ICD-9 diagnoses in past 6 months, 6) chronic pain, and 7) self-rated health status.

We trained 4 machine learning models (logistic regression, multilayer perceptron, SVM, and random forest) by using the 7 selected predictors. On the basis of the 10-fold cross-validation results, we chose logistic regression as the ultimate PreDICD model because it outperformed the other 3 models in AUROC (logistic regression = 0.81, multilayer perceptron = 0.80, SVM = 0.73, random forest = 0.78). The logistic regression model also had the highest percentage of correctly classified instances of depression (logistic regression = 74.0%, multilayer perceptron = 73.5%, SVM = 71.6%, random forest = 72.6%) and sensitivity (logistic regression = 0.65, multilayer perceptron = 0.55, SVM = 0.61, random forest = 0.65), and the second highest specificity (logistic regression = 0.81, multilayer perceptron = 0.88, SVM = 0.80, random forest = 0.79) among the 4 models.

The predictors of depression used for the PreDICD model are listed in Table 2. The results show that the following 5 predictors collectively increased the likelihood that the patient would be depressed: female (odds ratio [OR] = 2.35, *P* < .001), total number of complications from diabetes (OR = 1.35, *P* < .001), a history of major depressive disorder (OR = 4.03, *P* < .001), number of comorbidities, measured by the number of ICD-9 diagnoses in previous 6 months (OR = 1.03, *P* = .04), and chronic pain (OR = 2.13 *P* < .001). Two predictors decreased the likelihood that the patient would be depressed: good diabetes self-care, measured by Toobert diabetes self-care (OR = 0.66, *P* < .001), and self-rated good health status (OR = 0.45, *P* < .001).

**Table 2:** Ultimate PreDICD Model^a^: Predictors of Depression Among Patients with Diabetes

Predictor	Estimate (SE)	Odds Ratio (95% Confidence Interval)	*P* Value
Female	0.86 (0.13)	2.35 (1.83–3.03)	<.001
Toobert diabetes self-care	−0.42 (0.04)	0.66 (0.61–0.72)	<.001
Total number of diabetes complications	0.30 (0.06)	1.35 (1.21–1.51)	<.001
History of major depressive disorder	1.39 (0.21)	4.03 (2.66–6.10)	<.001
Number of ICD-9 diagnoses in past 6 months	0.03 (0.01)	1.03 (1.00–1.06)	.04
Chronic pain	0.75 (0.13)	2.13 (1.61–2.74)	<.001
Self-rated health status	−0.81 (0.08)	0.45 (0.38–0.52)	<.001

Abbreviations: ICD-9, *International Classification of Diseases, 9th Revision*; PreDICD, Predicting Diabetes Patients with Comorbid Depression; SE, Standard Error

a Logistic regression model: N = 1,776, estimate of intercept = 1.635, Ridge parameter for avoiding overfitting and improving predictive ability = 10^−10^.

### Evaluating and comparing the model-based depression screening policy

The policy that assigns 2-step PHQ screening only to patients predicted by the PreDICD model as being depressed was compared with mass screening and with 3 heuristic-based partial screening policies. Results (Table 3) show that, compared with mass screening, the model-based policy can save resources and time; specifically, the policy reduces the proportion of patients receiving PHQ-2 screening from 100% to 32.3%, the proportion of patients receiving PHQ-9 screening from 29.1% to 16.5%, and the number of screening questions asked per patient from about 4 to 1.8. However, the model-based policy is also shown to decrease the rate of depression identification from about 80% to 50%.

**Table 3:** Comparison of Model-Based Depression Screening Policy with Other Screening Policies

Measure	Model-Based Policy^a^^,^ b	Mass Screening^a^		Heuristic-Based Partial Screening Policy^a^					
				No. 1^c^		No. 2^d^		No. 3^e^	
	Value	Value	*P* f	Value	*P* f	Value	*P* f	Value	*P* f
**Scenario 1: 2-step PHQ screening^g^**									
Proportion of patients receiving PHQ-2 screening	32.3	100	<.001	8.6	<.001	52.4	<.001	56.2	<.001
Proportion of patients receiving PHQ-9 screening	16.5	29.1	<.001	5.5	<.001	16.9	0.726	19.2	.007
Depression identification rate	49.5	78.7	<.001	18.5	<.001	46.4	0.372	53.8	.15
Number of screening questions asked per patient	1.80	4.04	<.001	0.56	<.001	2.23	<.001	2.47	<.001
**Scenario 2: complete PHQ-9 screening^h^**									
Proportion of patients receiving PHQ-9 screening	32.3	100	<.001	8.6	<.001	52.4	<.001	56.2	<.001
Depression identification rate	62.9	100	<.001	20.6	<.001	58.6	0.247	67.3	.21
Number of screening questions asked per patient	2.91	9.00	<.001	0.77	<.001	4.72	<.001	5.06	<.001

Abbreviations: PHQ, Patient Health Questionnaire; PHQ-2, Patient Health Questionnaire, 2 items; PHQ-9, Patient Health Questionnaire, 9 items; PreDICD, Predicting Diabetes Patients with Comorbid Depression.

a Values are percentages unless otherwise indicated.

b Model-based policy: assigning 2-step PHQ screening or full PHQ-9 screening to patients predicted by the PreDICD model as being depressed.

c Heuristic-based partial screening policy no.1: assigning 2-step PHQ screening or full PHQ-9 screening to patients with previous diagnosis with major depressive disorder.

d Heuristic-based partial screening policy no. 2: assigning 2-step PHQ screening or full PHQ-9 screening to patients with severe diabetes (hemoglobin A1c ≥9%).

e Heuristic-based partial screening policy no. 3: assigning 2-step PHQ screening or full PHQ-9 screening to patients with either previous diagnosis with major depressive disorder or severe diabetes (hemoglobin A1c ≥9%).

f McNemar’s test for paired dichotomous variables for comparing proportion of patients receiving PHQ-2 screening, proportion of patients receiving PHQ-9 screening and depression identification rate, and paired *t* test for comparing number of screening questions asked per patient.

g Patients who meet screening policy inclusion criteria are evaluated using the 2-step PHQ screening (ie, PHQ-2 is first assigned, and then patients with PHQ-2 score3 are further evaluated by PHQ-9).

h Complete PHQ-9 screening is assigned for all patients who meet screening policy inclusion criteria.

The heuristic-based policy that assigned 2-step PHQ screening to patients with a previous diagnosis of major depressive disorder could identify only about 20% of depressed patients. Compared with the model-based policy, the other 2 heuristic-based policies had insignificantly different rates of depression identification but cost significantly more in provider resources and time.

A comparison of the model-based depression screening policy using 1-step PHQ-9 with mass PHQ-9 screening (Table 3) revealed that the model-based policy saved provider resources and time; specifically, the policy reduced the proportion of patients receiving PHQ-9 screening from 100% to 32.3% and the number of screening questions asked per patient from about 9 to 2.9. The rate of depression identification, however, decreases from 100% to about 63%. The heuristic-based policy that assigns PHQ-9 screening to patients with a previous diagnosis of major depressive disorder had a low (20.6%) depression identification rate. Similar to the results for 2-step PHQ screening, the other 2 heuristic-based policies had insignificantly different rates of depression identification but cost significantly more in provider resources and time compared with the model-based policy.

## Discussion

The PreDICD study developed a clinical forecasting model predicting the occurrence of depression among patients with diabetes by using data from 2 clinical trials. The study considered 20 candidate predictors and compared 4 machine learning models: logistic regression, multilayer perceptron, SVM, and random forest. The ultimate PreDICD model is logistic regression, with 7 predictors in the model: 1) female, 2) Toobert diabetes self-care, 3) total number of diabetes complications, 4) previous diagnosis of major depressive disorder, 5) number of ICD-9 diagnoses in previous 6 months, 6) presence of chronic pain, and 7) self-rated health status. Five of the 7 predictors typically can be acquired from EMR: female sex, total number of diabetes complications, previous diagnosis of major depressive disorder (ICD-9 diagnosis codes 296.2 and 296.3), number of ICD-9 diagnoses in previous 6 months, and presence of chronic pain (ICD-9 diagnosis code 338.2). Diabetes treatment guidelines recommend that health care providers collect data on 2 of the predictors during clinic visits: Toobert diabetes self-care scale, because most of the day-to-day care inherent in diabetes is handled by patients or their families (24), and self-rated health status, because it is strongly correlated with clinical outcomes such as mortality (25).

Three prior studies also predicted the occurrence of depression on the basis of health-related data. King et al (13) developed a model that forecasts depression diagnosed by DSM-IV major depression criteria from prospectively collected data from Europe and Chile; and Wang et al (14) developed a similar prediction model by using data from a US national survey. Huang et al (15) developed a prediction model for depression, measured by PHQ-9, from the EMR of a health system. The PreDICD model has comparable predictive ability (AUROC = 0.81) to those 3 studies (AUROC = 0.75–0.85). However, we emphasize that the predictive ability of those studies cannot be easily compared because they either focused on different patient populations or used different depression measures as the outcome.

The model-based screening policy that assigns depression screening only to patients predicted as being depressed by the PreDICD model can improve efficiency in identifying depressed patients with diabetes compared with mass screening (ie, saving about 50% to 60% of provider resources and time at the price of missing identification of about 30% of patients with depression). Such a finding is an encouraging step toward implementing a decision-support system based on available medical information that allows providers to better prioritize the use of resources and time.

As health delivery systems increasingly take on responsibility for managing population health, model-based screening can help providers reach out to patients who are identified as at-risk by the model. For example, the National Committee for Quality Assurance’s standard requires patient-centered medical homes to provide depression screening (26). The PreDICD model-based policy could establish a preliminary screening step for medical homes to routinely survey patients and target high-risk patients, especially nonengaged ones, for depression screening. Our findings also suggest that providers should refrain from using heuristic-based screening policies that assign depression screening to patients with diabetes and a history of depression, severe diabetes, or both, because those policies either have low rates of depression identification or higher cost in provider resources and time than the model-based policy.

This study has several limitations. The PreDICD model combines 2 data sets with somewhat different populations recruited at different times and does not account for possible cohort and period effects on the health conditions of the study populations. Study patients were predominantly Hispanics from the safety-net population with diabetes, which may limit the generalizability of the PreDICD model to wider patient populations because underlying determinants of depression may differ by racial/ethnic group (27). Culling available medical information introduces limitations, including limitations on accuracy and completeness of ICD-9 codes, and the total number of diabetes complications. Another limitation is that 2 of the 7 predictors, Toobert diabetes self-care scores and self-rated health status, despite recommendations, are not currently available in many medical practices. This could reduce the benefit from using the model-based policy if practitioners need to expend additional effort to collect information for those predictors. However, an Institute of Medicine committee recommended ways to cull EMR to capture social and behavioral determinants of health (28). If this recommendation is implemented, information availability may not be a barrier to adopting the model-based policy.

Future work should validate and refine the PreDICD model for broader patient populations to improve its generalizability. Also, research to extend the PreDICD model from predicting current depression to forecasting future depression could help health care providers to identify patients with diabetes who are at high future risk of depression and thus warrant repeated depression screening. The model could alternatively be extended from single-level to multilevel logistic regression to account for possible cohort and period effects, and thus improve the model’s predictive ability. The model should also be tested in a clinical environment to verify the feasibility of implementing a decision-support system and to evaluate its influences on clinical outcomes and operations, including costs and cost-savings. Finally, the machine learning methods demonstrated in the study can be applied to predicting clinical outcomes related to other conditions and could be useful in future research initiatives, such as the National Institutes of Health’s recently launched Precision Medicine Initiative (29).

Our PreDICD study developed a prediction model with compelling predictive ability for forecasting comorbid depression among patients with diabetes. Adopting such a model-based policy has the potential to outperform other heuristic approaches by better assisting health care providers to increase efficiency in managing their patients with depression and better prioritize the use of their resources and time to deliver effective care for high-risk patients.
